# Incidence and associated factors of developing second pelvic malignant neoplasms among prostate cancer patients treated with radiotherapy

**DOI:** 10.3389/fonc.2023.1260325

**Published:** 2023-11-17

**Authors:** Youbiao Wang, Ru Chen, Xinxi Deng, Xinghua Jiang

**Affiliations:** ^1^ Department of Urology, The Second People’s Hospital of Jingdezhen City, Jingdezhen, Jiangxi, China; ^2^ Department of Urology, The First Affiliated Hospital of Nanchang University, Nanchang, Jiangxi, China; ^3^ Department of Urology, Jiujiang First People’s Hospital, Jiujiang, Jiangxi, China

**Keywords:** prostate cancer, pelvic malignant neoplasms, bladder cancer, rectal cancer, radiotherapy, SEER

## Abstract

**Objective:**

To identify risk factors of secondary pelvic malignant neoplasms (SPMNs) among prostate cancer (PCa) patients treated with radiotherapy. Simultaneously, population-based data were used to validate the high risk of SPMNs in PCa patients with radiotherapy.

**Materials and methods:**

We identified male patients diagnosed with PCa (localized and regional) as the first primary cancer and pelvic malignant neoplasm (including bladder and rectal cancer) as secondary cancer from Surveillance, Epidemiology, and End Results database (1975-2020). An external validation cohort was obtained from the First Affiliated Hospital of Nanchang University. The Fine-Gray competing risk regression and Poisson regression were utilized to evaluate the risk of SPMNs development. Poisson regression was also performed to calculate the standardized incidence ratio (SIR). The Kaplan-Meier method was used to assess the overall survival (OS) of patients with SPMNs.

**Results:**

89397 PCa patients treated with radiotherapy were enrolled. We identified associated factors of SPMNs, including age at diagnosis, race, year of diagnosis, marital status, radiation strategy and latency. In the multivariable competing risk regression model and Poisson regression model, a significantly higher risk of SPMNs development was observed in patients over 50 years(P<0.05), white patients(P<0.001), unmarried patients and treated with brachytherapy combined with external beam radiotherapy or brachytherapy(P<0.05). Patients treated with radiotherapy had a higher bladder and rectal cancer incidence than the general population. Patients who developed SPMNs showed poorer OS.

**Conclusion:**

We identified several risk factors associated with SPMNs and confirmed a relatively higher incidence of bladder and rectal cancer among PCa patients with radiotherapy. These results help tailor treatment and surveillance strategies.

## 1 Introduction

It is estimated that in 2019, nearly three million men in the United States were newly diagnosed with prostate cancer (PCa) (1). PCa was the most prevalent of all male cancers. While early-stage diagnosis and treatment have proven successful in managing PCa, reducing long-term mortality and improving quality of life remains a top priority. However, it is important to note that the high prevalence of PCa may be in part due to observation-based management techniques as well as the widespread use of PSA testing (2).

Radiotherapy (RT) and radical prostatectomy (RP) are standard treatment options for active treatment of localized PCa, and there is evidence that they have similar long-term disease-free survival rates (3). However, observational data with low to moderate risk of bias indicate that radiotherapy may be associated with a higher risk of overall and prostate cancer-specific mortality when compared with surgery (4). A particularly worrying potential effect of prostate radiotherapy is radiation-induced second malignancy. There have been many previous studies on the risk analysis of secondary malignant neoplasm (SMN) for PCa after radiotherapy, but the results were not consistent (1, 3, 5–7). The results of most studies suggested that prostate irradiation increased the risk of developing secondary pelvic malignant neoplasms (SPMNs), which included bladder cancer (BC) and rectum cancer (RC). A retrospective study suggested that PCa patients who received RT were more prone to developing a second primary cancer compared to those who did not receive the therapy, with a higher risk over time. Despite a lower incidence and risk of second primary cancer (8). A recent meta-analysis to evaluate the second malignancies after radiotherapy for PCa suggested that radiotherapy was associated with an increased risk of secondary BC and RC compared with patients who did not treat with radiotherapy (7). However, most of the controversy was that the included studies are retrospective, so the reliability of the results was still limited. In theory, large prospective studies aimed at minimizing the effects of possible confounding factors would address the real risk of SMN after prostate irradiation. However, it seems unlikely that such trials will be carried out in the near future for logistical reasons.

One hypothesis based on this study is that radiotherapy for PCa will increase the risk of developing secondary pelvic neoplasms incorporating BC and RC. This study intended to explore relevant risk factors of SPMNs development for PCa patients treated with radiotherapy using contemporary data in a large population-based cohort. In addition, we used a ratio which was expressed with standardized incidence ratio (SIR) to evaluate the risk of SPMNs development in PCa patients treated with radiotherapy and without.

## 2 Methods

### 2.1 Database and study population

**Figure 1:**
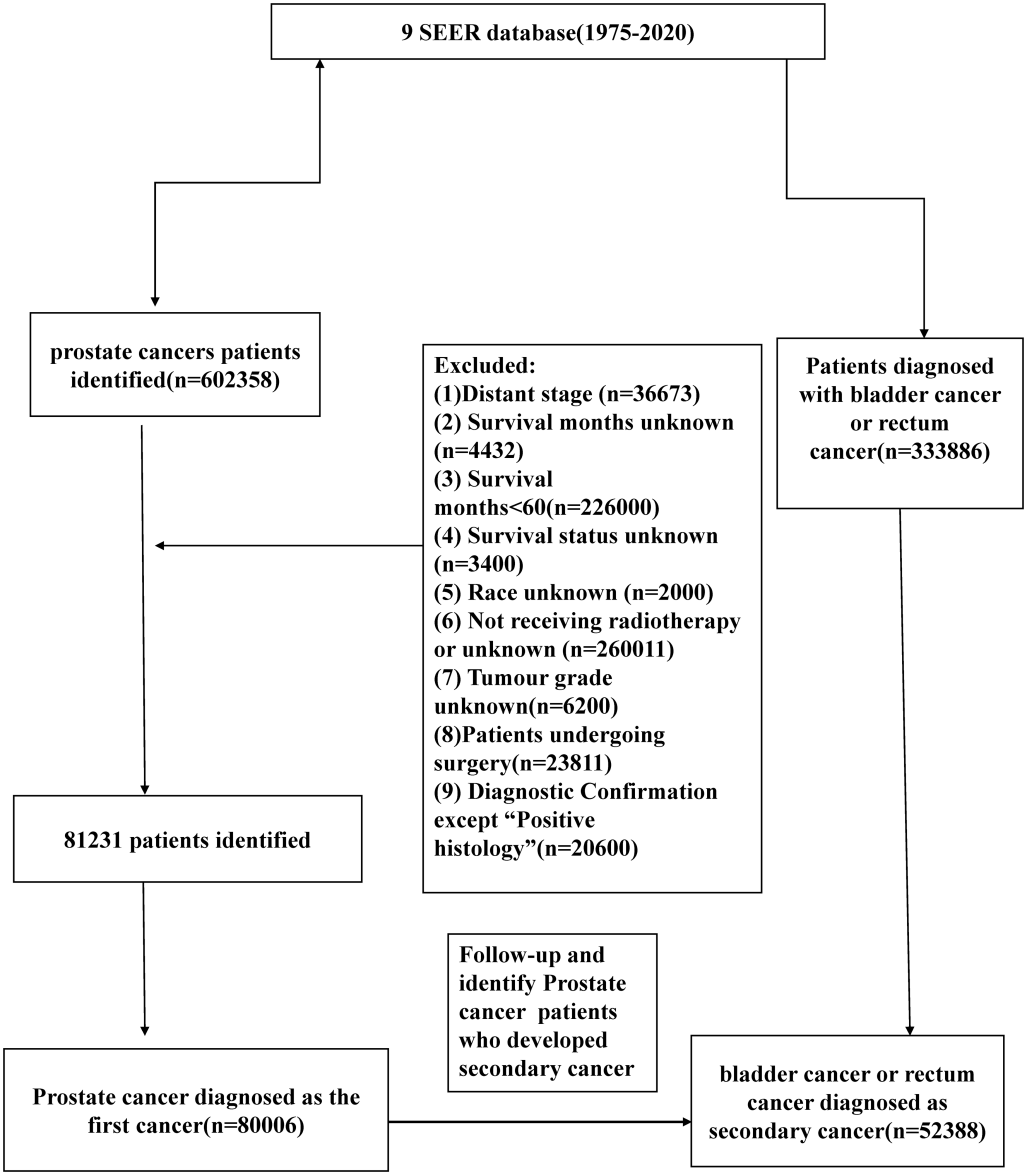
Flow-chart showing the procedure used to identify male patients diagnosed with prostate cancer as the first primary cancer bladder cancer (BC) and rectal cancer (RC) as secondary cancers from the Surveillance, Epidemiology and End Results (SEER) database (1975-2018; 9 registries).

We identified male patients diagnosed with prostate cancer as the first primary cancer from the Surveillance, Epidemiology and End Results (SEER) database (1975-2020; 9 registries) (**Figure 1**). Then, we created a study population that included cases diagnosed with bladder cancer (BC) and rectal cancer (RC) as secondary cancers from the same database. All cancer sites were identified based on the case list of “*The International Classification of Diseases for Oncology, Third Edition (Site recode ICD-O-3)*”. We enrolled PCa patients who received radiotherapy only and excluded patients undergoing surgery. The diagnostic confirmation method was restricted to positive histology. The other exclusion criteria are as follows: (1) tumor stage as distant; (2) survival months unknown;(3) survival months less than 60 months (4) survival status unknown; (5) Race unknown; (6) tumor grade unknown, (7) Diagnosis after 2013 year.

We have obtained an external validation cohort consisting of patients diagnosed with prostate cancer at the First Affiliated Hospital of Nanchang University between 2010 and 2019. These patients received surgery or RT. Demographic and clinical data, including age, marital status, smoking habits, alcohol consumption, Prostate-Specific Antigen (PSA) levels, distant metastasis, and the number of patients observed with bladder or rectal cancer after treatment, were collected.

### 2.2 Variable definition

The following data were collected: age at diagnosis (<50 years, 50-70 years, >70 years); race [white, black, others (American/Indian/Alaska/Native and Asian/Pacific Islander)]; year of diagnosis, marital status (married, unmarried, unknown); Gleason biopsy (6–10); clinical T stage (cT1, cT2, cT3, cT4, unknown); AJCC Stage Group (II, III, IV, unstaged); summary stage (localized, regional, unknown); tumor grade (grade I or grade II, grade III or grade IV); radiation strategy [external beam radiotherapy(EBRT); external beam radiotherapy-brachytherapy (EBRT+BT); brachytherapy(BT)]; survival month; survival status.

### 2.3 Definition and follow-up of SPMNs

The primary outcome of this study was the development of an SPMN, which was defined as bladder cancer or rectal cancer occurring more than 5 years after PCa patients received radiotherapy in consideration of the incubation period of at least 5 years from radiation exposure to a solid tumor (9). The SEER program followed the guidelines of *the third edition of the International Classification of Oncology Diseases* to distinguish between SPMN and recurrent diseases. The cancer history is obtained according to the “ Sequence number “ case list, which lists the order in which all the primary tumors can be reported in the patient’s life. The follow-up for SPMN began 5 years after PCa diagnosis and ended at the date of diagnosis of BC or RC, all-cause death, or the last follow-up, whichever occurred first. The last follow-up data was December 31, 2020.

### 2.4 Statistical analysis

Fine-Gray competing risk regression analysis was utilized to calculate the cumulative incidence of SPMN development. Experiencing end of follow-up or death from all-cause were considered competing events. The multivariable competing risk model was established by using a backward selection procedure with variables which were statistically significant in the univariable analyses.

The results were presented as hazard ratios (HRs) and 95%CIs. Poisson regression analysis was used to estimate the association between different incorporated factors and the risk of SPMN development, and the results exhibited as SPMN associated risk and 95%CIs. The missing values were imputed using multiple imputation method for conducting sensitivity analysis. Meanwhile, Poisson regression analysis was also performed to calculate the standardized incidence ratio (SIR) and 95%CIs. The SIR was defined as a ratio of the observed SPMN incidence rate in PCa survivors to the pelvic malignant neoplasm (PMN) incidence rate in the U.S. general population. The SIRs were calculated with SEER*Stat 8.4.0(ID: 20420-Nov2020). Then, we calculated the SIRs stratified by year of diagnosis, age at diagnosis, and latency to furtherly evaluate the incidence of SPMNs associated with radiotherapy.

To evaluate the prognosis of SPMNs, survival analyses were performed with the Kaplan–Meier method and log-rank tests to calculate the overall survival (OS) for patients who developed SPMNs and with only primary PCa. The only primary PCa was defined as a patient who had only been diagnosed with PCa and had no other cancer throughout his lifetime. Propensity score matching (PSM) was performed to adjust the potential baseline matched 1:1 for survival comparison (caliper set at 0.02).

All statistical analyses were performed by R software (version 4.1.3). P-values less than 0.05 were statistically significant.

## 3 Results

### 3.1 Patient characteristics

89397 PCa patients who received radiotherapy were enrolled in our study. There were 2125(2.38%) PCa survivors who developed SPMN, and there were 1758(1.97%) cases who developed bladder cancer and 367(0.41%) developed rectal cancer, respectively (**Table 1**).

**Table 1:** Baseline characteristics of patients with prostate cancer treated with radiotherapy.

	No. (%)
Characteristic	Radiotherapy (n= 80006)
Age at diagnosis	
<50 years	1752(2.19%)
50-70 years	43795(54.74%)
>70 years	34459(43.07%)
Race	
White	62404(78%)
black	12112(15.14%)
others^1^	5490(6.86%)
Year of diagnosis	
1995-1999	16305(20.38%)
2000-2004	24538(30.67%)
2005-2009	24105(30.13%)
2010-2014	15058(18.82%)
Marital status	
Married	16121(20.15%)
Single	37521(46.89%)
Widowed/Divorced	20051(25.07%)
Unknown	6313(7.89%)
Gleason biopsy	
6	4040(5.05%)
7	7872(9.84%)
8-10	2776(3.47%)
Unknown	65318(81.64%)
Clinical T stage	
cT1	26762(33.45%)
cT2	15817(19.77%)
cT3	1280(1.6%)
cT4	96(0.12%)
Unknown	36051(45.06%)
AJCC Stage Group	
II	40651(50.81%)
III	1920(2.4%)
IV	400(0.5%)
Unstage	37035(46.29%)
Lymph node status	
N0	40083(50.1%)
Nx	12800(16%)
unknown	27123(33.9%)
Summary stage	
Localized	67413(84.26%)
Regional	4208(5.26%)
Unknown	8385(7.48%)
Grade	
Grade I or Grade II	49283(61.6%)
Grade III or Grade IV	30723(38.4%)
Radiation strategy	
EBRT	44259(55.32%)
EBRT+BT	14705(18.38%)
BT	21042(26.3%)
**Developed SPMN**	2125(2.38%)
**Developed BC**	1758(1.97%)
**Developed RC**	367(0.41%)

EBRT, external beam radiotherapy; EBRT+BT, interstitial brachytherapy or a combination of external beam radiotherapy; BT, brachytherapy; SPMN, second pelvic malignant neoplasm; BC, bladder cancer; RC, rectal cancer; AJCC, American Joint Committee on Cancer.

Other: American/Indian/Alaska/Native and Asian/Pacific Islander.

We finally identified 116 PCa patients who received surgery (67, 57.75%) and RT (49, 42.25%), respectively from the First Affiliated Hospital of Nanchang University (**Supplementary Table 1**). During the follow-up period, we observed 3 cases of BC or RC among patients in the surgery group and 9 cases among patients in the radiation therapy group(P=0.001).

### 3.2 Cumulative incidences of SPMNs

**Figure 2:**
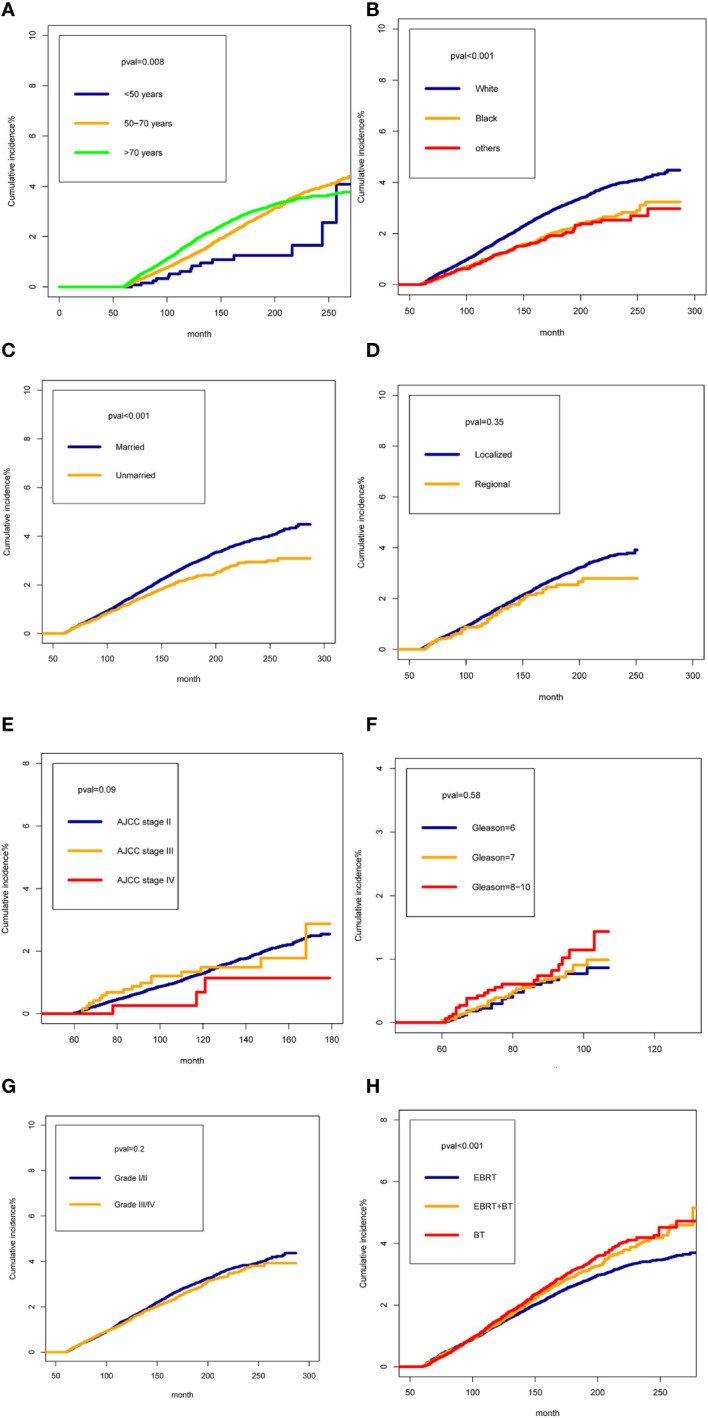
The cumulative incidences of Second Pelvic Malignant Neoplasm in prostate cancer (PCa) patients treated with radiotherapy by different characteristics: **(A)**: for age; **(B)** for race; **(C)** for marital status **(D)** for tumor stage; **(E)** for AJCC Stage Group; **(F)** for Gleason biopsy; **(G)** for tumor grade; **(H)** radiotherapy strategy.

We presented the cumulative incidences of SPMNs in PCa patients treated with radiotherapy by different characteristics. The cumulative incidences of SPMNs in patients aged less than 50 years were significantly lower than in patients aged 50-70 years and more than 70 years (**Figure 2A**) (P=0.008). For different race patients, white patients showed a higher cumulative incidence than black and other races (**Figure 2B**) (P<0.001). Married patients had a statistically higher incidence than those unmarried (**Figure 2C**) (P<0.001). We did not obtain statistical differences in cumulative incidences distribution when stratifying the study population by summary stage, AJCC stage, Gleason biopsy and tumor grade (**Figures 2D–G**) (all P>0.05). For patients who received different radiotherapy strategy, we observed a relatively lower cumulative incidence of SPMNs in patients who received EBRT when compared with those with EBRT+BT or BT (**Figure 2H**) (P<0.001).

### 3.3 Risk factors associated with SPMNs

We performed competing risk regression and Poisson regression to identify risk factors associated with SPMNs development. We found statistically significant variables, including age at diagnosis, race, year of diagnosis, marital status, tumor grade, radiation strategy and latency in the univariate competing risk regression and univariate Poisson regression (**Table 2**). In the multivariable competing risk regression model, a significantly higher risk of SPMNs development was observed in 50-70 years (HR:1.78,95%CI:1.16- 2.73) and >70 years (HR: 2.05,95%CI: 1.33- 3.15) patients compared with those age less than 50 years. White patients showed a relatively higher risk than black and other patients (P<0.001). Patients diagnosed after 2000 exhibited a descending risk compared with those diagnosed between 1995-1999 (P<0.001). Unmarried PCa patients had a relatively higher risk than married cases (HR: 1.19; 95%CI: 1.08-1.31). Patients who received EBRT+BT or BT showed a higher risk in comparison to patients with EBRT (HR: 1.15; 95%CI: 1.04-1.26 for EBRT+BT; HR: 1.16; 95%CI: 1.06-1.26 for BT). For patients with latency more than 10 years, they showed a significantly higher risk than patients who had survival time between 5 and 10 years (P<0.001). After using the multiple imputation technique to fill in the missing values, similar results were obtained as before. **Supplementary Table 2** recorded In the multivariable Poisson regression, we similarly identified the statistically significant variables associated with SPMNs development, including age at diagnosis, race, year of diagnosis, marital status, radiation strategy and latency.

**Table 2:** Risk factors of developing SPMN (bladder cancer or rectum cancer) after prostate cancer diagnosis among patients receiving radiotherapy by Statistical Method.

Characteristic	Competing risk regression				Poisson regression			
	Univariate analysis HR (95% CI)	P-value	Multivariable analysis HR (95% CI)	P-value	Univariate analysis HR (95% CI)	P-value	Multivariable analysis HR (95% CI)	P-value
Age at diagnosis								
<50 years	Ref.		Ref.		Ref.		Ref.	
50-70 years	1.89(1.23-2.90)	0.014^※^	1.78(1.16- 2.73)	0.027^※^	1.97(1.32-3.12)	0.009^※^	1.83(1.22-2.9)	0.02^※^
>70 years	2.08(1.35-3.18)	0.005^※^	2.05(1.33- 3.15)	<0.001^※^	2.32(1.56-3.68)	0.001^※^	2.18(1.45-3.46)	0.02^※^
Race								
White	Ref.		Ref.		Ref.		Ref.	
black	0.68(0.61-0.77)	<0.001^※^	0.75(0.66-0.84)	<0.001^※^	0.63(0.56-0.71)	<0.001^※^	0.75(0.66-0.84)	<0.001^※^
others^1^	0.66(0.55-0.78)	<0.001^※^	0.67(0.57-0.79)	<0.001^※^	0.65(0.55-0.77)	<0.001^※^	0.67(0.56-0.79)	<0.001^※^
Year of diagnosis								
1995-1999	Ref.		Ref.		Ref.		Ref.	
2000-2004	0.86(0.79-0.93)	<0.001^※^	0.83(0.75-0.9)	<0.001^※^	0.79(0.68-0.9)	<0.001^※^	0.77(0.7-0.85)	<0.001^※^
2005-2009	0.69(0.63-0.76)	<0.001^※^	0.72(0.64-0.81)	<0.001^※^	0.43(0.39-0.48)	<0.001^※^	0.5(0.45-0.56)	<0.001^※^
2010-2014	0.67(0.56-0.8)	<0.001^※^	0.96(0.78-0.99)	<0.001^※^	0.16(0.13- 0.18)	<0.001^※^	0.27(0.22-0.33)	<0.001^※^
Marital status								
Married	Ref.		Ref.		Ref.		Ref.	
Unmarried	1.29(1.17-1.42)	<0.001^※^	1.19(1.08-1.31)	0.004^※^	1.34(1.22-1.48)	<0.001^※^	1.18(1.07-1.3)	0.005^※^
Gleason biopsy								
6	Ref.				Ref.			
7	1.03(0.7-1.5)	0.89			0.99(0.68- 1.47)	0.98		
8-10	1.2(0.76-1.9)	0.53			1.29(0.81-2.03)	0.35		
AJCC Stage Group								
II	Ref.				Ref.			
III	0.95(0.63-1.4)	0.83			0.96(0.62-1.4)	0.86		
IV	0.47(0.18-1.2)	0.19			0.48(0.15-1.08)	0.2		
Summary stage								
Localized	Ref.				Ref.			
Regional	0.83(0.66-1.04)	0.18			0.84(0.66-1.04)	0.19		
Grade								
Grade I or Grade II	Ref.		Ref.		Ref.			
Grade III or Grade IV	0.85(0.79-0.92)	<0.001^※^	0.99(0.91-1.07)	0.83	0.61(0.57-0.66)	<0.001^※^	0.98(0.9-1.07)	0.768
Radiation strategy								
EBRT	Ref.		Ref.		Ref.		Ref.	
EBRT+BT	1.14(1.04-1.25)	0.023^※^	1.15(1.04-1.26)	0.02^※^	1.17(1.07-1.29)	0.004^※^	1.14(1.03-1.25)	0.02^※^
BT	1.17(1.07-1.27)	0.002^※^	1.16(1.06-1.26)	0.005^※^	1.15(1.05-1.24)	0.007^※^	1.14(1.04-1.24)	0.01^※^
Latency								
5-10 years	Ref.		Ref.		Ref.			
11-15 years	1.47(1.34-1.62)	<0.001^※^	1.55(1.39-1.72)	<0.001^※^	2.31(2.1-2.55)	<0.001^※^	1.76(1.59-1.97)	<0.001^※^
16-20 years	1.61(1.46-1.78)	<0.001^※^	1.57(1.39-1.76)	<0.001^※^	3.38(3.06-3.75)	<0.001^※^	2.02(1.8-2.28)	<0.001^※^
21-25 years	1.6(1.38-1.86)	<0.001^※^	1.43(1.2-1.69)	<0.001^※^	3.89(3.32-4.54)	<0.001^※^	2.04(1.7-2.43)	<0.001^※^

EBRT, external beam radiotherapy; EBRT+BT, interstitial brachytherapy or a combination of external beam radiotherapy; BT, brachytherapy; SPMN, second pelvic malignant neoplasm; AJCC, American Joint Committee on Cancer; HR, hazard ratio; CI, confidence interval.

^1^Other: American/Indian/Alaska/Native and Asian/Pacific Islander.

^※^ : statistical significance.

### 3.4 Dynamic incidence evaluation for SPMNs

We evaluated the SIRs of BC and RC for PCa patients treated with radiotherapy and patients undergoing surgery, respectively (**Table 3**). For PCa patients treated with radiotherapy, we observed a significantly increased incidence of BC and RC compared with the US general population (SIR: 1.44; 95%CI: 1.38- 1.5; P<0.05 for BC; SIR: 1.48; 95%CI: 1.35- 1.62; P<0.05 for RC). In the subgroup analysis by year of diagnosis, we found the increasing incidence of BC after 1995 and incidence of RC after 2000. In analyses of SIRs for different age, a relatively higher incidence than US general population was observed in PCa patients over 60 years old for BC and in PCa patients over 70 years old for RC. For PCa patients who survived longer than 5 years, we obtained a significantly increased incidence of BC and RC. For patients undergoing surgery, no increased incidences of BC and RC were observed in PCa patients when compared with US general population. Similar results were obtained when we stratified the PCa patients by year of diagnosis and latency. We just observed the increasing incidences of BC in PCa patients aged 60-64 years and 65-69 years.

**Table 3:** Standardized incidence ratio of bladder cancer and rectum cancer after prostate cancer diagnosis among patients receiving radiotherapy or without radiotherapy.

	Bladder cancer		Rectum cancer	
	Adjusted SIR (95% CI)	P-value	Adjusted SIR (95% CI)	P-value
RT vs US general population				
**All patients**	1.44(1.38- 1.50)	<0.05	1.48(1.35- 1.62)	<0.05
Year of diagnosis				
1995-1999	1.37(1.18-1.57)	<0.05	1.21(0.90-1.58)	NS
2000-2004	1.44(1.29-1.60)	<0.05	1.32(1.05-1.65)	<0.05
2005-2009	1.34(1.21-1.48)	<0.05	1.58(1.28-1.93)	<0.05
2010-2015	1.54(1.41-1.68)	<0.05	1.49(1.19-1.85)	<0.05
Age at diagnosis				
<60 years	NA	NA	NA	NA
60-64 years	2.08(1.44-2.91)	<0.05	1.20(0.55-2.27)	NS
65-69 years	1.72(1.41-2.07)	<0.05	1.45(0.98-2.06)	NS
70-74 years	1.33(1.16- 1.52)	<0.05	1.35(1.03-1.73)	<0.05
75-79 years	1.36(1.24-1.49)	<0.05	1.44(1.18-1.74)	<0.05
80-84 years	1.38(1.27-1.50)	<0.05	1.47(1.22-1.75)	<0.05
85+ years	1.53(1.42-1.65)	<0.05	1.66(1.38-1.98)	<0.05
Survival months				
5-10 years	1.27(1.19-1.35)	<0.05	1.18(1.02-1.35)	<0.05
>10 years	1.61(1.52-1.70)	<0.05	1.87(1.64-2.11)	<0.05
Undergoing surgery vs US general population				
**All patients**	1.00(0.98-1.02)	NS	0.92(0.89-0.95)	<0.05
Year of diagnosis				
1995-1999	0.92(0.86- 0.97)	<0.05	0.93(0.84- 1.02)	NS
2000-2004	0.98(0.93-1.03)	NS	0.92(0.84-1.01)	NS
2005-2009	1.03(0.99- 1.08)	NS	0.90(0.83- 0.98)	NS
2010-2015	1.00(0.96- 1.04)	NS	0.97(0.89- 1.05)	NS
Age at diagnosis				
<60 years	NA	NA	NA	NA
60-64 years	1.32(1.22- 1.42)	<0.05	0.98(0.87- 1.11)	NS
65-69 years	1.07(1.01-1.14)	<0.05	0.85(0.77- 0.94)	<0.05
70-74 years	0.97(0.93-1.02)	NS	0.88(0.81-0.96)	<0.05
75-79 years	0.96(0.92-1.01)	NS	0.90(0.83-0.98)	<0.05
80-84 years	0.95(0.91- 0.99)	<0.05	0.85(0.78-0.92)	<0.05
85+ years	0.94(0.91- 0.98)	<0.05	0.94(0.87-1.01)	NS
Survival months				
5-10 years	1.00(0.98-1.02)	NS	0.97(0.92-1.02)	NS
>10 years	0.96(0.94- 0.99)	<0.05	0.88(0.84- 0.93)	<0.05

SIR, standardized incidence ratio; RT, radiotherapy; NA, not applicable; NS: no significance.

### 3.5 Survival outcome of SPMNs

We compared the OS of PCa patients with only primary tumor and patients who developed SPMNs to evaluate the prognosis of SPMNs. Survival curves revealed that patients with only PCa had a significantly better OS than those who developed SPMNs for PCa patients with a survival time longer than 140 months (**Supplementary Figure 1A**). This difference in survival outcome became more prominent after PSM (**Supplementary Figure 1B**). In addition, we assessed the prognosis of BC or RC development, respectively. We failed to obtain a statistical difference in OS between patients with only primary tumor and developed BC (**Supplementary Figure 1C**). However, patients with BC development showed poorer survival outcomes than those without after PSM(P<0.001). Patients with only PCa had a significantly better OS than patients with RC development before and after PSM (**Supplementary Figures 1D–F**).

## 4 Discussion

In this study, we identified several associated factors of SPMNs development, including age at diagnosis, race, year of diagnosis, marital status, radiation strategy, and latency in PCa patients treated with radiotherapy by multiple statistical methods. Meanwhile, we found a higher incidence of subsequent BC and RC in PCa with radiotherapy than in the general US population, and no increase in the incidence of SPMNs was observed in PCa undergoing surgery. Finally, we observed a relatively poorer OS in PCa patients who developed SPMNs when compared with those with only primary PCa.

Normal aging had been reported as a risk factor for secondary malignancies by many previous studies (10). For example, ANURAG K et al. found increasing age was accompanied by a risk of BC development for patients diagnosed with PCa as first cancer (11). Moreover, for the general population, old age has also been proved to be an essential factor in the occurrence of BC and RC (12, 13). Our study also obtained similar results and identified a higher risk of SPMNs development among older PCa patients treated with radiotherapy. Meanwhile, older patients may have lower sensitivity to radiation therapy and often require higher radiation doses during the radiotherapy process. Older prostate cancer patients typically have more traditional cancer risk factors, such as familial inheritance, overeating, smoking, etc. These risk factors may have a composite effect, leading to an increase in the incidence of other cancers. These results suggested that the surveillance of SPMNs in elderly patients with PCa receiving radiotherapy might be a more practical method. An interesting finding is a decrease in risk of SPMNs development in PCa patients diagnosed after 2000 years. This might be due to the decline in the age of diagnosis caused by PSA screening and early detection of preclinical cancer (10). It was consistent with the lower incidence of SPMNs in the young PCa patient. Another similar result was a significant increase in the risk of SPMNs development in patients with PCa who had survived longer than 10 years. The possible explanation for this result was that a longer survival time might increase the exposure probability of carcinogens (14, 15). It is not easy to make a reasonable explanation for the risk difference of SPMNs development between different races and marital status. The possible explanation for this phenomenon is discrepancies in living habits (including smoking), living environment, carcinogens exposure, and radiation sensitivity (2, 14). For example, married individuals are more likely to adopt healthy lifestyle habits, such as maintaining a regular diet and exercise routine, undergoing regular check-ups, and practicing smoking cessation and alcohol moderation. These healthy habits may potentially reduce the risk of developing cancer. At the same time, certain racial groups may face more economic pressures and disadvantages in society, including low income, limited health insurance coverage, and fewer medical resources. This may result in late cancer diagnosis and fewer treatment options, thereby increasing the risk of developing other types of cancer. However, because we lacked this information and were limited by retrospective studies, more relevant evidence was needed to explain it in the future.

Notably, we observed a relatively lower risk of SPMNs development in patients with EBRT than in those with EBRT+BT or BT. This result was not consistent with some previous results. For instance, Moon et al. observed an increased risk of BC after EBRT compared with brachytherapy, and the data was also from the SEER database (16). Generally, intensity modulated radiotherapy and charged particle therapy might reduce the risk of SMNs by reducing the number of tissues exposed to high doses of radiation (15). Kishan J. Pithadia et al. based on SEER found patients treated with intensity-modulated radiotherapy (IMRT) had no significant differences with those with three-dimensional conformal radiation therapy (3DCRT) (17). However, the radiation dose of radioactive implants to the pelvis was still relatively high, even higher than EBRT to some extent (18). Therefore, it was no surprise that an increased incidence of SPMNs was shown in PCa patients treated with EBRT+BT or BT. Meanwhile, considering the differences in the definition of SPMN, the choice of the incubation period, the length of follow-up, the methods of the cohort population and the sample size between studies, we might need more studies to explain these inconsistent results.

Although there had been studies focusing on the association between radiotherapy and SPMNs, the conclusions were conflicting (2, 3, 10). Considering that the patient selection bias caused by the retrospective study might affect the reliability of the results partly, we introduce epidemiological indicators (SIR) to evaluate the difference in subsequent SPMNs development among PCa patients treated with radiotherapy and without. This kind of data based on a large population will be more representative for most US population. Meanwhile, because the calculation of SIR is based on the data observed by the population, this index reduces the artificial bias to a great extent (19). In addition, we took the incidence of SPMNs in the general population as a control, while previous studies mainly had compared the risk of SPMNs development among PCa patients in two cohorts, including patients who received radiotherapy and prostatectomy. We think that setting the incidence outside our study population as a comparison might make our results more convincing (20).

It is no surprise that PCa patients with subsequent SPMNs showed a poorer OS than those with only primary PCa because the prognosis of PCa was significantly better than BC and RC, and the 5-year relative survival rate of local or regional PCa approached 100% after aggressive treatment (1). Notably, our study began five years after the diagnosis of PCa, not at the time of SPMNs diagnosis. Therefore, this difference in survival was not significant during the initial study period but became more prominent with the extension of follow-up time. Recent research findings demonstrate that even for low/medium risk patients, those undergoing ultra-conformal hypofractionated RT face a heightened risk of mortality from second cancers, surpassing the risk posed by prostate cancer itself (21). These findings serve as a crucial reminder for physicians to exercise greater caution during the evaluation of treatment necessities for prostate cancer patients, taking into account the potential risks associated with developing a secondary malignancy. In the case of patients with mild or low-risk disease presentations, prioritizing the avoidance of unnecessary treatment becomes paramount in order to minimize the potential for additional health hazards.

The strengths of this study include a long follow-up period to discover potential SPMNs as well as the utilization of a large, population-based database, which allowed for the application of the results across the USA. In addition, we used external cohort data to validate our results, thus increasing the scientific validity of our results. However, our study was not devoid of limitations. First, we tried to include all available factors in our research for analysis. Still, due to the limitations of the database, we lacked some crucial factors like smoking, lifestyle, genetic background, psychosocial factors, more detail information of tumor stage and Gleason score, and radiation dose to prevent us from adjusting our analysis to understand the potential effect of these confounding factors (14). Secondly, considering PCa patients with secondary RC were insufficient for further investigation, we set our positive event in this study as SPMNs development. Then, we excluded patients with PCa for whom radiotherapy information was unknown, and this population represented a larger number of patients in the overall study population, which may to some extent undercut the validity of our results. Last but not least, because of the limitations of the SEER database, specific information on radiotherapy, such as more detailed radiotherapy modalities, is lacking to obtain more detailed information on outcomes. Still, we think this effect on our conclusions was negligible.

## 5 Conclusion

This study comprehensively evaluated the risk factors of SPMNs development in PCa patients receiving radiotherapy. In addition, we demonstrated a high incidence of SPMNs in PCa patients treated with radiotherapy by comparing with the general population. Increased risk of BC or RC in PCa patients with radiotherapy might have implications for public health, cancer surveillance and patient counseling. Perhaps most importantly, the study confirmed the belief that for patients with low-risk prostate cancer who did not need treatment at all, a second malignant tumor should be added to the already long list of avoidable risks associated with treatment.

## Data availability statement

The original contributions presented in the study are included in the article/**Supplementary Material**. Further inquiries can be directed to the corresponding authors.

## Ethics statement

Ethical review and approval was not required for the study on human participants in accordance with the local legislation and institutional requirements. Written informed consent from the participants was not required to participate in this study in accordance with the national legislation and the institutional requirements.

## Author contributions

YW: Writing – original draft, Writing – review & editing. RC: Writing – original draft. XD: Data curation, Formal Analysis, Investigation, Writing – review & editing. XJ: Methodology, Writing – original draft.

## Supplementary material

Supplementary Figure 1 Overall survival between patients with only prostate cancer and developing Second Pelvic Malignant Neoplasm(SPMN): **(A)** all SPMNs before PSM; **(B)** all SPMNs after PSM; **(C)** For bladder cancer before PSM; **(D)** For bladder cancer after PSM **(E)** For rectal cancer before PSM; **(F)** For rectal cancer after PSM.
