# Supplementary figures and images for: Incidence and associated factors of developing second pelvic malignant neoplasms among prostate cancer patients treated with radiotherapy
Source: Front Oncol. 2023 Nov 17;13:1260325. doi: 10.3389/fonc.2023.1260325 (PMC10693410; doi:10.3389/fonc.2023.1260325)

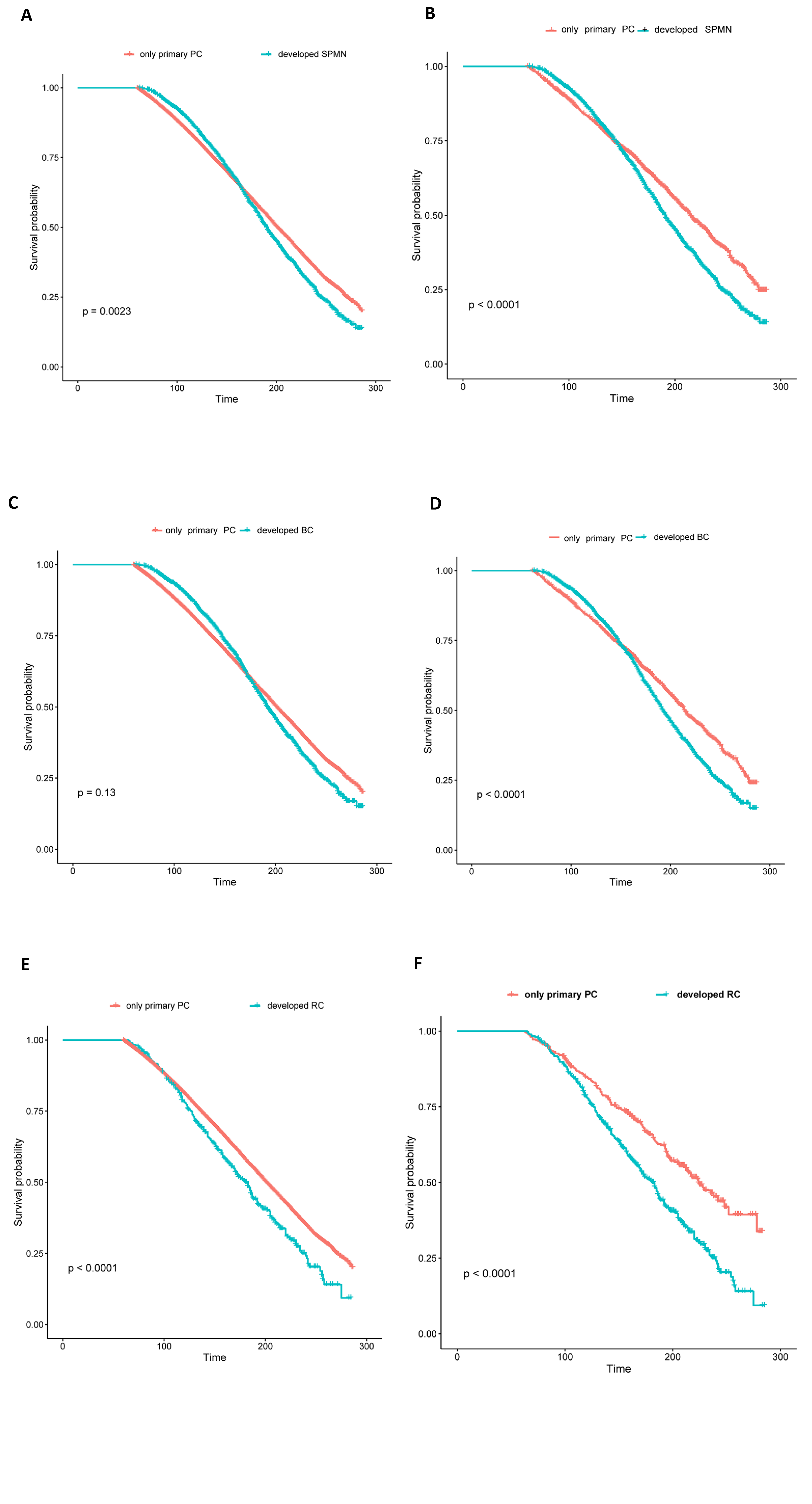

Supplement: Supplementary Figure 1 — Overall survival between patients with only prostate cancer and developing Second Pelvic Malignant Neoplasm(SPMN): (A) all SPMNs before PSM; (B) all SPMNs after PSM; (C) For bladder cancer before PSM; (D) For bladder cancer after PSM (E) For rectal cancer before PSM; (F) For rectal cancer after PSM. [file Image_1.tif]
