# Supplementary material for: Incidence and associated factors of developing second pelvic malignant neoplasms among prostate cancer patients treated with radiotherapy
Source: Front Oncol. 2023 Nov 17;13:1260325. doi: 10.3389/fonc.2023.1260325 (PMC10693410; doi:10.3389/fonc.2023.1260325)
Supplement: Supplementary file 2 [file Table_1.docx]

| **Supplement table 1:** **Basic characteristics of prostate cancer patients treated with surgery or radiotherapy in the First Affiliated Hospital of Nanchang University** | | | |
| --- | --- | --- | --- |
|  | **Surgery** | **Radiotherapy** | **P value** |
| **Overall** | 67(57.75%) | 49(42.25%) |  |
| **Age(year)** |  |  |  |
| mean | 59.08 | 61.25 | 0.44 |
| median | 57 | 57 |  |
| **Marital status** |  |  | 0.56 |
| No | 44(65.7%) | 16(34.0%) |  |
| Yes | 23(34.3%) | 33(66.0%) |  |
| **Smoking** |  |  | 0.001 |
| No | 29(44.7%) | 38(79.5%) |  |
| Yes | 38(55.3%) | 11(20.5%) |  |
| **Drinking** |  |  | <0.001 |
| No | 26(38.3%) | 46(94.9%) |  |
| Yes | 41(61.7%) | 3(5.1%) |  |
| **Metastases** |  |  | 0.568 |
| No | 48 (72.3%) | 32(66.7%) |  |
| Yes | 19(27.7%) | 17(33.3%) |  |
| **PSA** |  |  | 0.675 |
| ≤6 | 35(53.2%) | 21(43.6%) |  |
| 7 | 12(19.1%) | 11(23.1%) |  |
| ≥8 | 20(27.7%) | 17(33.3%) |  |
| **Number of patients observed BC or RC** | 3(4.47%) | 9(18.36%) | 0.001 |
| BC: bladder cancer; RC: rectal cancer | | | |
