# Supplementary material for: Incidence and associated factors of developing second pelvic malignant neoplasms among prostate cancer patients treated with radiotherapy
Source: Front Oncol. 2023 Nov 17;13:1260325. doi: 10.3389/fonc.2023.1260325 (PMC10693410; doi:10.3389/fonc.2023.1260325)
Supplement: Supplementary file 3 [file Table_2.docx]

| **Supplement table 2: Risk factors of developing SPMN (bladder cancer or rectum cancer) after prostate cancer diagnosis (<5 years) among patients receiving radiotherapy by Statistical Method** | | | | | | | | |
| --- | --- | --- | --- | --- | --- | --- | --- | --- |
| **Characteristic** | **Competing risk regression** | | | | **Poisson regression** | | | |
|  | **Univariate analysis**  **HR (95% CI)** | **P-value** | **Multivariable analysis**  **HR (95% CI)** | **P-value** | **Univariate analysis**  **HR (95% CI)** | **P-value** | **Multivariable analysis**  **HR (95% CI)** | **P-value** |
| **Age at diagnosis** |  |  |  |  |  |  |  |  |
| <50 years | Ref. |  | Ref. |  | Ref. |  | Ref. |  |
| 50-70 years | 1.69(1.03-2.70) | 0.01^※^ | 1.79 (1.17- 2.79) | 0.02^※^ | 1.67(1.02-3.02) | 0.001^※^ | 1.81(1.12-2.8) | 0.01^※^ |
| >70 years | 2.18(1.45-3.48) | 0.001^※^ | 2.05(1.33- 3.15) | <0.001^※^ | 2.12(1.36-3.48) | 0.001^※^ | 2.11(1.41-3.42) | 0.01^※^ |
| **Race** |  |  |  |  |  |  |  |  |
| White | Ref. |  | Ref. |  | Ref. |  | Ref. |  |
| black | 0.68(0.60-0.71) | <0.001^※^ | 0.75(0.61-0.82) | <0.001^※^ | 0.69(0.55-0.73) | <0.001^※^ | 0.65(0.61-0.74) | <0.001^※^ |
| others^1^ | 0.66(0.55-0.76) | <0.001^※^ | 0.67(0.51-0.81) | <0.001^※^ | 0.55(0.51-0.67) | <0.001^※^ | 0.61(0.51-0.72) | <0.001^※^ |
| **Year of diagnosis** |  |  |  |  |  |  |  |  |
| 1995-1999 | Ref. |  | Ref. |  | Ref. |  | Ref. |  |
| 2000-2004 | 0.66(0.59-0.73) | <0.001^※^ | 0.82(0.75-0.9) | <0.001^※^ | 0.77(0.67-0.9) | <0.001^※^ | 0.67(0.7-0.85) | <0.001^※^ |
| 2005-2009 | 0.61(0.53-0.72) | <0.001^※^ | 0.73(0.61-0.81) | <0.001^※^ | 0.53(0.39-0.48) | <0.001^※^ | 0.51(0.45-0.56) | <0.001^※^ |
| 2010-2014 | 0.61(0.56-0.81) | <0.001^※^ | 0.96(0.88-0.99) | <0.001^※^ | 0.16(0.13- 0.18) | <0.001^※^ | 0.26(0.22-0.33) | <0.001^※^ |
| **Marital status** |  |  |  |  |  |  |  |  |
| Married | Ref. |  | Ref. |  | Ref. |  | Ref. |  |
| Unmarried | 1.19(1.17-1.32) | <0.001^※^ | 1.19(1.08-1.32) | 0.001^※^ | 1.24(1.22-1.48) | <0.001^※^ | 1.18(1.03-1.3) | 0.004^※^ |
| **Gleason biopsy** |  |  |  |  |  |  |  |  |
| 6 | Ref. |  |  |  | Ref. |  |  |  |
| 7 | 1.03(0.71-1.54) | 0.89 |  |  | 0.99(0.78- 1.47) | 0.98 |  |  |
| 8-10 | 1.22(0.76-1.91) | 0.53 |  |  | 1.29(0.91-2.03) | 0.35 |  |  |
| **AJCC Stage Group** |  |  |  |  |  |  |  |  |
| Ⅱ | Ref. |  |  |  | Ref. |  |  |  |
| Ⅲ | 0.95(0.63-1.4) | 0.83 |  |  | 0.96(0.62-1.4) | 0.86 |  |  |
| Ⅳ | 0.47(0.18-1.2) | 0.2 |  |  | 0.48(0.15-1.08) | 0.21 |  |  |
| **Summary stage** |  |  |  |  |  |  |  |  |
| Localized | Ref. |  |  |  | Ref. |  |  |  |
| Regional | 0.83(0.56-1.04) | 0.19 |  |  | 0.84(0.66-1.14) | 0.2 |  |  |
| **Grade** |  |  |  |  |  |  |  |  |
| Grade I or Grade II | Ref. |  | Ref. |  | Ref. |  |  |  |
| Grade III or Grade IV | 0.85(0.79-0.95) | <0.001^※^ | 0.98(0.91-1.17) | 0.84 | 0.61(0.57-0.66) | <0.001^※^ | 0.98(0.9-1.17) | 0.76 |
| **Radiation strategy** |  |  |  |  |  |  |  |  |
| EBRT | Ref. |  | Ref. |  | Ref. |  | Ref. |  |
| EBRT+BT | 1.11(1.04-1.15) | 0.003^※^ | 1.17(1.04-1.21) | 0.01^※^ | 1.16(1.07-1.29) | 0.001^※^ | 1.12(1.03-1.25) | 0.01^※^ |
| BT | 1.16(1.07-1.27) | 0.001^※^ | 1.16(1.06-1.26) | 0.001^※^ | 1.17(1.04-1.24) | 0.002^※^ | 1.16(1.04-1.24) | 0.01^※^ |
| **Latency** |  |  |  |  |  |  |  |  |
| 5-10 years | Ref. |  | Ref. |  | Ref. |  |  |  |
| 11-15 years | 1.27(1.14-1.42) | <0.001^※^ | 1.51(1.34-1.62) | <0.001^※^ | 2.21(2.1-2.45) | <0.001^※^ | 1.72(1.69-1.97) | <0.001^※^ |
| 16-20 years | 1.6(1.47-1.76) | <0.001^※^ | 1.51(1.39-1.86) | <0.001^※^ | 3.28(3.06-3.85) | <0.001^※^ | 2.12(1.8-2.38) | <0.001^※^ |
| 21-25 years | 1.6(1.38-1.86) | <0.001^※^ | 1.41(1.21-1.59) | <0.001^※^ | 3.49(3.22-4.54) | <0.001^※^ | 2.14(1.7-2.33) | <0.001^※^ |
| EBRT: external beam radiotherapy; EBRT+BT: interstitial brachytherapy or a combination of external beam radiotherapy; BT: brachytherapy; SPMN: second pelvic malignant neoplasm; AJCC: American Joint Committee on Cancer; HR: hazard ratio; CI: confidence interval.  ^1^Other: American/Indian/Alaska/Native and Asian/Pacific Islander | | | | | | | | |
